# Family resilience and social support as mediators of caregiver burden and capacity in stroke caregivers: a cross-sectional study

**DOI:** 10.3389/fpsyg.2024.1435867

**Published:** 2024-12-03

**Authors:** Qihang Xu, Jingjing Ma, Yiqing Zhang, Jiehua Gan

**Affiliations:** ^1^Department of Pharmacy, Ningbo Medical Center LiHuili Hospital, Ningbo, China; ^2^Department of Nursing, Ningbo Medical Center LiHuili Hospital, Ningbo, China; ^3^Department of Neurology, Ningbo Medical Center LiHuili Hospital, Ningbo, China

**Keywords:** stroke, family, resilience, caregivers, burden, capacity, cross-sectional survey, nursing stroke

## Abstract

**Introduction:**

Caring for stroke survivors poses significant challenges to survivors and caregivers; understanding these relationships can inform targeted interventions and support systems.

**Aim:**

This study investigates the influencing factors of caregiver burden and the potential mediating roles of family resilience and perceived social support between caregiver burden and caregiver capacity.

**Design:**

This is a cross-sectional study.

**Methods:**

The participants in this study included 462 stroke survivors and their primary caregivers from Ningbo Lihuili Hospital in Zhejiang Province, China. Primary caregivers completed several assessments, including the Shortened Chinese Version of the Family Resilience Assessment Scale (FRAS), the Perceived Social Support Scale (PSSS), the Zarit Caregiver Burden Interview (ZCBI), and the Family Caregiver Task Inventory (FCTI). The ZCBI was used to evaluate caregiver burden, while the activities of daily living (ADL) were assessed for stroke survivors to measure their actual level of dependence. The mediating effect of family resilience was estimated using the bootstrap method through Jamovi version 2.3.0 with the mediation plug-in, while controlling for sociodemographic variables.

**Results:**

The results showed that caregiver burden was influenced by stroke survivors’ level of dependence on activities of daily living (ADL), caregiver education level, caregiver health status, and average daily care time. The family resilience mediated the relationship between caregiver burden and caregiver capacity (*b* = 0.141; 95% confidence interval: 0.091 to 0.176). Additionally, perceived social support mediated the relationship between caregiver burden and caregiver capacity (*b* = 0.080; 95% confidence interval: 0.041 to 0.110).

**Conclusion:**

These findings indicate that enhancing family resilience and perceived social support can be strategies for alleviating caregiver burden. Clinical practitioners should actively assess the family resilience and social support of stroke survivors, and implement interventions that promote resilience and strengthen social support, thereby reducing caregiver burden and improving the health outcomes of stroke survivors.

## Introduction

Stroke is a significant global health issue, ranking as the second leading cause of long-term disability worldwide. Approximately 15 million new stroke cases occur globally each year, and about 5 million people survive a stroke but face long-term disabilities (Potter et al., 2022). In China alone, nearly 2,000,000 individuals are affected by stroke each year (Wu et al., 2019). Unfortunately, the burden of stroke is expected to increase further with China’s aging population (Tu et al., 2023).

Stroke survivors often face chronic disability that not only affects the physical and cognitive abilities of the survivors but also significantly impacts their emotional well-being and overall quality of life (Yang et al., 2016). Caring for stroke survivors is a complex and demanding task that often falls on the shoulders of family caregivers. However, the demands of caregiving can lead to significant caregiver burden, with studies indicating that over 50% of family caregivers experience high levels of burden, resulting in emotional exhaustion, physical fatigue, and negative mental health outcomes (Riffin et al., 2019).

There is a well-established relationship between caregiver burden and caregiver capacity (Pugh et al., 2022; van Roij et al., 2021). Caregiver competence refers to the ability of caregivers to effectively meet the physical, emotional, and social needs of stroke survivors while maintaining their own well-being (van Roij et al., 2021). This competence encompasses the knowledge, skills, resources, and support systems available to caregivers. Research indicates that increased caregiver burden can negatively impact caregivers’ ability to provide care, ultimately affecting the recovery and health outcomes of stroke survivors (Cho et al., 2020; Tan et al., 2023).

Family resilience is a critical concept defining a family’s ability to adapt, recover, and flourish when facing challenges. It encompasses aspects of the family unit such as cohesion, effective communication, problem-solving skills, and overall fortitude in navigating the intricacies of caregiving responsibilities (Fredrickson, 2001; Lee et al., 2023). Family resilience plays a significant role in enhancing caregivers’ adaptability when faced with the challenges of caregiving. Studies conducted in populations of cancer patients and children have shown that family resilience can reduce caregiver burden (Cui et al., 2023; Park et al., 2022). Additionally, research indicates that family resilience enhances caregiving capacity (Zhong et al., 2022). However, there is limited research exploring the potential moderating role of family resilience in the relationship between stroke caregiver capacity and caregiver burden, highlighting the need for further investigation (Zhang et al., 2023).

Perceived social support refers to an individual’s subjective assessment of the assistance received from family, friends, neighbors, and other social networks. Caregivers who perceive a robust support system often experience reduced emotional exhaustion and anxiety, enabling them to maintain a healthier mental state and greater motivation during caregiving (Zhang and Dong, 2022). Studies have shown that higher levels of perceived social support can significantly diminish caregiver burden and enhance caregiver competence (Da Costa et al., 2023; Stawnychy et al., 2021). However, the role of perceived social support as a mediating factor in the context of stroke caregiving remains underexplored, warranting further investigation (Zhong et al., 2020).

In China, it is traditional for family members to take responsibility for the care of stroke survivors (Mou et al., 2023). Once a patient has a stroke, they are typically admitted to the neurology department of a general hospital on an emergency basis. After 10 to 15 days of acute treatment, the survivors is then transferred to a rehabilitation ward for a period of 3 to 6 months. During this time, many survivors rely heavily on their families for long-term care. The primary caregiver is tasked with managing the dietary and physical needs of the stroke survivor and remains as a 24-h companion in the hospital, unable to return home to attend to other family responsibilities. Consequently, the concept of caregiver burden is multifaceted and encompasses the physical, psychosocial, and financial toll of providing care (Cheng et al., 2018). Family caregivers face numerous challenges in providing essential care and support to their loved ones.

he study is based on the Stress-Resource Balance Model. When individuals face stressors (Hobfoll, 1989), such as caregiving burden, the negative effects of that stress can be mitigated if they have access to sufficient resources, such as family resilience and social support. These resources encompass not only external social support but also adaptive resources within the family (Thoits, 2011).

Therefore, this study explores the influencing factors of caregiver burden and the potential mediating roles of family resilience and perceived social support in the relationship between caregiver burden and caregiver capacity among family caregivers of stroke survivorss. We hypothesize that higher levels of family resilience and perceived social support will be associated with lower levels of caregiver burden. By identifying the mediating roles of these factors, we can gain a deeper understanding of the complex dynamics involved in stroke caregiving and inform the development of interventions and support systems that effectively address the needs of caregivers and promote their well-being.

## Methods/design

### Participants

This cross-sectional study was conducted in Ningbo, China, from March to September 2023. Dyads consisting of hospitalized stroke survivors and their primary family caregivers were voluntarily recruited from a public hospital in Ningbo Province. Participants were voluntarily recruited through the following methods: (1) Hospital Announcements: We placed announcements in the hospital’s common areas, including waiting rooms and patient wards, inviting participants in the study. (2) Doctor Referrals: Medical staff, including physicians and nurses, were informed about the study and encouraged to refer eligible participants. Inclusion criteria for participants were: (1) A confirmed diagnosis of stroke; (2) Both stroke survivors and caregivers were above 18 years old; (3) The primary family caregiver during hospitalization was a willing family member who provided primary care for the patient. Exclusion criteria were: (1) severe complications or caregivers with severe physical illnesses; (2) a history of depression or current use of antidepressant or psychotropic medications; (3) refusal to participate or unavailability during the survey. Recommended sample sizes for general multilayer models in previous guidelines are 30, 50, 100, and 200. For dyad studies, a sample size of more than 50 pairs is suggested to obtain reliable and valid estimates (Du and Wang, 2016). To maximize the sample size while minimizing repetition, a total sample size of 400 was set, aiming for a required sample of 460 dyads after considering a 15% invalid questionnaire rate.

### Measures

During the hospitalization of stroke survivors and their primary family caregivers, the survey was conducted by trained assessors. Assessors underwent training covering the study’s purpose and ethics, questionnaire content, and communication skills to effectively engage with participants. Participants were asked to complete a structured questionnaire, which typically took around 30 min. The questionnaire included five sections, covering various aspects such as demographic characteristics, activities of daily living, family resilience, caregiver burden, caregiver capacity, and perceived social support. Data collection took place in a quiet, private environment within the hospital to enhance participant comfort.

1. The demographic data collected in the survey covered a range of factors, such as gender, age, education level, time of diagnosis, employment status, medical payment methods, and income.
2. The activities of daily living (ADL) scoring assessment was conducted to evaluate the level of functional independence in various daily activities, such as personal hygiene, dressing, eating, toileting, transferring, and walking (Mlinac and Feng, 2016). Scores on the ADL scale ranged from 0 to 100, with higher scores indicating a higher degree of independence. Individuals with scores ranging from 99 to 61 were considered partially independent, meaning they could independently complete some activities but may require occasional assistance. Scores between 60 and 41 indicated a moderate level of dependence, where significant assistance was needed to perform daily activities. A score of 40 indicated severe dependence, meaning the individual was unable to complete most activities without full assistance or care from others. A nurse carried out this ADL assessment during the hospitalization period.
3. We utilized the Zarit Burden Interview (ZBI), which consists of 22 items, to assess caregiver burden. Respondents were asked to rate their level of burden on a five-point Likert scale. Higher scores on the ZBI indicate higher levels of burden experienced by caregivers. The ZBI has demonstrated good reliability and validity in its English and Chinese versions. In previous studies, the English version showed a Cronbach’s alpha of 0.90–0.92 for reliability and a validity range of 0.40–0.80 (Lu et al., 2009; Zarit et al., 1980). Similarly, the Chinese version demonstrated a Cronbach’s alpha of 0.88–0.94 for reliability and a validity range of 0.67–0.77. In our study, the Cronbach’s alpha for the ZBI was calculated to be 0.94.
4. We employed the Family Resilience Scale, which consists of 32 items to assess family resilience. These items are divided into three subscales: family communication and problem-solving, utilizing social resources, and maintaining a positive attitude. Each item was rated on a four-point Likert scale, ranging from 1 (strongly disagree) to 4 (strongly agree). The total score on the scale ranged from 32 to 128, with higher scores indicating higher levels of family resilience. The reliability and validity of the Family Resilience Scale have been established in both English and Chinese versions. In previous studies, the English version demonstrated a Cronbach’s alpha of 0.87–0.93 for reliability and a validity range of 0.69–0.85. Similarly, the Chinese version showed a Cronbach’s alpha of 0.88–0.96 for reliability and a validity range of 0.75–0.86 (Chow et al., 2022). In our study, the Cronbach’s alpha for the Family Resilience Scale was calculated to be 0.93.
5. The Family Caregiver Training and Intervention (FCTI) is a 25-item assessment tool that we used to measure caregiver capacity. This tool evaluates various aspects of caregiving, including responsiveness, adaptation to the caregiving role, emotional management, resource assessment, and lifestyle adjustments. Higher scores on the FCTI indicate lower caregiving capacity. The Chinese version of the FCTI, translated by Lee and Mok (2011) has shown good reliability with a Cronbach’s alpha of 0.93. In our study, the internal consistency of the FCTI was also excellent, with a Cronbach’s alpha of 0.93.
6. The Perceived Social Support Scale (PSSS) is a self-report measure developed by to evaluate an individual’s perception of social support from their social network. It consists of 12 items that capture different sources of social support, including family, friends, and significant others. Participants rate their level of agreement with each item on a scale ranging from 1 (very strongly disagree) to 7 (very strongly agree). Previous studies have consistently shown high internal consistency and reliability for the PSSS, with a Cronbach’s alpha coefficient of 0.914 (Liu et al., 2016). In the present study, the Cronbach’s alpha coefficient for the PSSS was also found to be 0.91. This study’s internal consistency was excellent, with a Cronbach‘s alpha 0.91.

### Outcome variable definition

Our analysis examined the relationships between caregiver capacity, family resilience, caregiver burden, and perceived social support. Caregiver capacity was the independent variable, while family resilience and perceived social support were the mediating variables; caregiver burden was the primary dependent variable of interest.

In order to identify the influencing factors on caregiver burden, we will also include sociodemographic characteristics and ADL scores as covariates in the analysis. In the mediation analysis, we take these covariates into account to control for potential confounding factors.

### Statistical analysis

In our data analysis, we utilized R language version 4.2.3. We employed descriptive statistics such as means, frequencies, and standard deviations to summarize the data. To examine the relationships between the variables of interest, we conducted various statistical tests, including one-way analysis of variance, linear regression analysis, and Pearson’s correlation coefficient (r). These tests allowed us to assess the associations and dependencies between the study variables. We used Jamovi version 2.3.0 with the mediation plug-in to explore the mediating role of family resilience. All statistical tests were conducted with a two-tailed approach, and statistical significance was determined using a *p*-value threshold of less than 0.05.

## Results

### Descriptive statistics

Of the 500 pairs of stroke survivors and family caregivers, 18 survivors refused to participate due to health problems, 12 caregivers declined due to a lack of interest, and eight were excluded from the questionnaire with missing data. The final sample included 462 survivor-caregiver dyads for a participation rate of 92.4%. Of the 462 valid questionnaires, the respondents comprised hospitalized stroke survivors and their primary caregivers. In the case of stroke survivors, the descriptive statistics are presented in Table 1. Most were male (237/462,51.3%), of whom 173/462 (37.45%) were in the 60–80 age group. Most respondents had an educational level of middle school and below (332/462, 71.86%). In the case of caregivers, descriptive statistics are displayed in Table 2. Most were female (256/462, 55.41%), with 203/462 (43.94%) aged between 18 and 40 years; 228 (49.35%) were stroke survivors children. The total length of care (months) included up to two weeks (317, 68.61%), 2–4 weeks (54, 11.69%), 4–5 weeks (47, 10.17%), and more than six weeks (44, 9.52%).

**Table 1:** Demographic characteristics of stroke survivors (*n* = 462).

(1) Stroke survivors demographics		*n* (%)
Gender	Male	237 (51.3)
	Female	225 (48.7)
Age (u)	18–40 years	48 (10.39)
	40–60 years	167 (36.15)
	60–80 years	173 (37.45)
	Over 80 years	74 (16.02)
Education	Middle school and below	332 (71.86)
	High school and college	102 (22.08)
	Bachelor’s degree or higher	28 (6.06)
Time of diagnosis	Less than 1 month ago	189 (40.91)
	1–2 months ago	123 (26.62)
	2–3 months ago	150 (32.47)
Employment status	Employed	126 (27.27)
	Resigned	61 (13.2)
	Retired	275 (59.52)
Medical payment	Urban medical insurance	129 (27.92)
	Rural medical insurance	195 (42.21)
	Self-financing	45 (9.74)
	Commercial insurance	93 (20.13)
Income (in RMB)	Less than 5,000 yuan	171 (37.01)
	5,000–7,000 yuan	152 (32.9)
	Over 7,000 yuan	139 (30.09)
Activities daily living	Independent	138 (29.87)
	Partially dependent	167 (36.15)
	Moderately dependent	112 (24.24)
	Severely dependent	45 (9.74)

**Table 2:** Demographic characteristics of primary stroke caregivers (*n* = 462).

(1) Stroke caregivers demographics		*n* (%)
Gender	Male	206 (44.59)
	Female	256 (55.41)
Age (years)	18–40	203 (43.94)
	40–60	130 (28.14)
	60–80	106 (22.94)
	Over 80	23 (4.98)
Education	Primary and below	58 (12.55)
	Middle school or equivalent	122 (26.41)
	High school and College	91 (19.7)
	Bachelor’s degree or higher	191 (41.34)
Caregiver place of residence	City	295 (63.85)
	Village	167 (36.15)
Employment status	Employed	255 (55.19)
	Resigned	57 (12.34)
	Retired	150 (32.47)
Familiarity with patient disease	Unfamiliar	58 (12.55)
	Familiar	248 (53.68)
	Very familiar	156 (33.77)
Caregiver-patient relationship	Spouse	139 (30.09)
	Children	228 (49.35)
	Parents	39 (8.44)
	Siblings of patients	44 (9.52)
	Other	12 (2.6)
Care experience	Yes	212 (45.89)
	No	250 (54.11)
Number of caregivers that families can share	Zero	95 (20.56)
	1 person	149 (32.25)
	2–3 persons	175 (37.88)
	More than 3 persons	43 (9.31)
Income (in RMB)	Less than 5,000 yuan	105 (22.73)
	5,000–7,000 yuan	158 (34.2)
	More than 7,000 yuan	199 (43.07)
Caregiver health status	Health	229 (49.57)
	Normal	211 (45.67)
	Poor	22 (4.76)
Total length of care (months)	Up to 2 weeks	317 (68.61)
	2–4 weeks	54 (11.69)
	4–5 weeks	47 (10.17)
	More than 6 weeks	44 (9.52)
Average daily care time	0–4 h	191 (41.34)
	5–8 h	165 (35.71)
	More than 8 h	106 (22.94)
Caregiver sleep	Good	46 (9.96)
	Normal	226 (48.92)
	Poor	190 (41.13)
Chronic disease	Yes	186 (40.26)
	No	276 (59.74)

### Factors affecting caregiver burden

The caregiver burden (Table 3) was found to be significantly influenced by the stroke survivors’ level of dependence on ADL and average daily care time. Specifically, the results revealed that being partially dependent (*β* = 0.23, *p* = 0.034), moderately dependent (*β* = 0.35, *p* = 0.004), or severely dependent (*β* = 0.42, *p* = 0.012) on ADL was positively correlated with caregiver burden. Caregivers with bachelor’s degrees or higher reported greater burdens (*β* = 0.51, *p* = 0.016) compared to those with primary education or below, while caregivers with normal health experienced less burden (*β* = −0.21, p = 0.016) compared to their healthy counterparts. Additionally, those providing care for more than 8 h a day reported lower burdens (*β* = −0.27, *p* = 0.049) than those caring for 0–4 h.

**Table 3:** Differences in caregiver burden by sociodemographic variables between stroke survivors and caregiver (*n* = 462).

Variables	*b*	S.E	*t*	*P*	*β* (95%CI)
**Stroke survivors ability daily living (ADL)**					
Independent					0.00 (Reference)
Partially dependent	0.23	0.11	2.13	0.034	0.23 (0.02 ~ 0.43)
Moderately dependent	0.35	0.12	2.91	0.004	0.35 (0.11 ~ 0.59)
Severely dependent	0.42	0.17	2.51	0.012	0.42 (0.09 ~ 0.74)
**Education of caregiver**					
Primary and below					0.00 (Reference)
Middle school or equivalent	0.26	0.17	1.52	0.130	0.26 (−0.08 ~ 0.59)
High school and college	0.23	0.18	1.30	0.193	0.23 (−0.12 ~ 0.58)
Bachelor’s degree or higher	0.51	0.21	2.41	0.016	0.51 (0.10 ~ 0.93)
Other	−0.17	0.29	−0.60	0.551	−0.17 (−0.73 ~ 0.39)
**Caregiver health status**					
Health					0.00 (Reference)
Normal	−0.21	0.09	−2.41	0.016	−0.21 (−0.37 ~ −0.04)
Poor	−0.03	0.20	−0.15	0.878	−0.03 (−0.43 ~ 0.37)
**Average daily care time**					
0–4 h					0.00 (Reference)
5–8 h	0.17	0.10	1.76	0.079	0.17 (−0.02 ~ 0.36)
More than 8 h	−0.27	0.13	−1.98	0.049	−0.27 (−0.53 ~ −0.01)

### The correlation matrix: resilience, burden, capacity, and social support

The correlation matrix (Table 4) revealed several associations among the FRAS-C, ZBI, FCTI, and PSSS scales. Higher levels of resilience, as measured by the FRAS-C scale, were negatively correlated with caregiver burden, as measured by the ZBI scale (*r* = −0.516, *p* < 0.01). Caregiver burden (ZBI scale) was positively correlated with lower caregiver capacity (FCTI scale), as indicated by the FCTI scale (*r* = 0.592, *p* < 0.01). Additionally, caregiver burden was negatively correlated with perceived social support, as measured by the PSSS scale (*r* = −0.425, *p* < 0.01). Moreover, the FCTI scale score negatively correlated with resilience and perceived social support. The FCTI scale showed negative correlations with the FRAS-C scale (*r* = −0.551, *p* < 0.01) and the PSSS scale (*r* = −0.405, *p* < 0.01).

**Table 4:** Correlations (*r*) between psychological burden, family resilience, and caregiver capacity.

	FRAS-C	ZBI	FCTI	PSSS
FRAS-C	1			
ZBI	−0.516**	1		
FCTI	−0.551**	0.592**	1	
PSSS	0.405**	−0.425**	−0.450**	1

The Zarit Burden Interview (ZBI) is used to evaluate caregiver burden.

The Family Resilience Scale (FRAS-C) assesses family resilience.

The Family Caregiver Task Inventory (FCTI) is used to evaluate the caregiver’s abilities.

**p-value < 0.01.

The Perceived Social Support Scale (PSSS) is a scale used to assess an individual’s perceived level of social support.

### Model test

**Figure 1:**
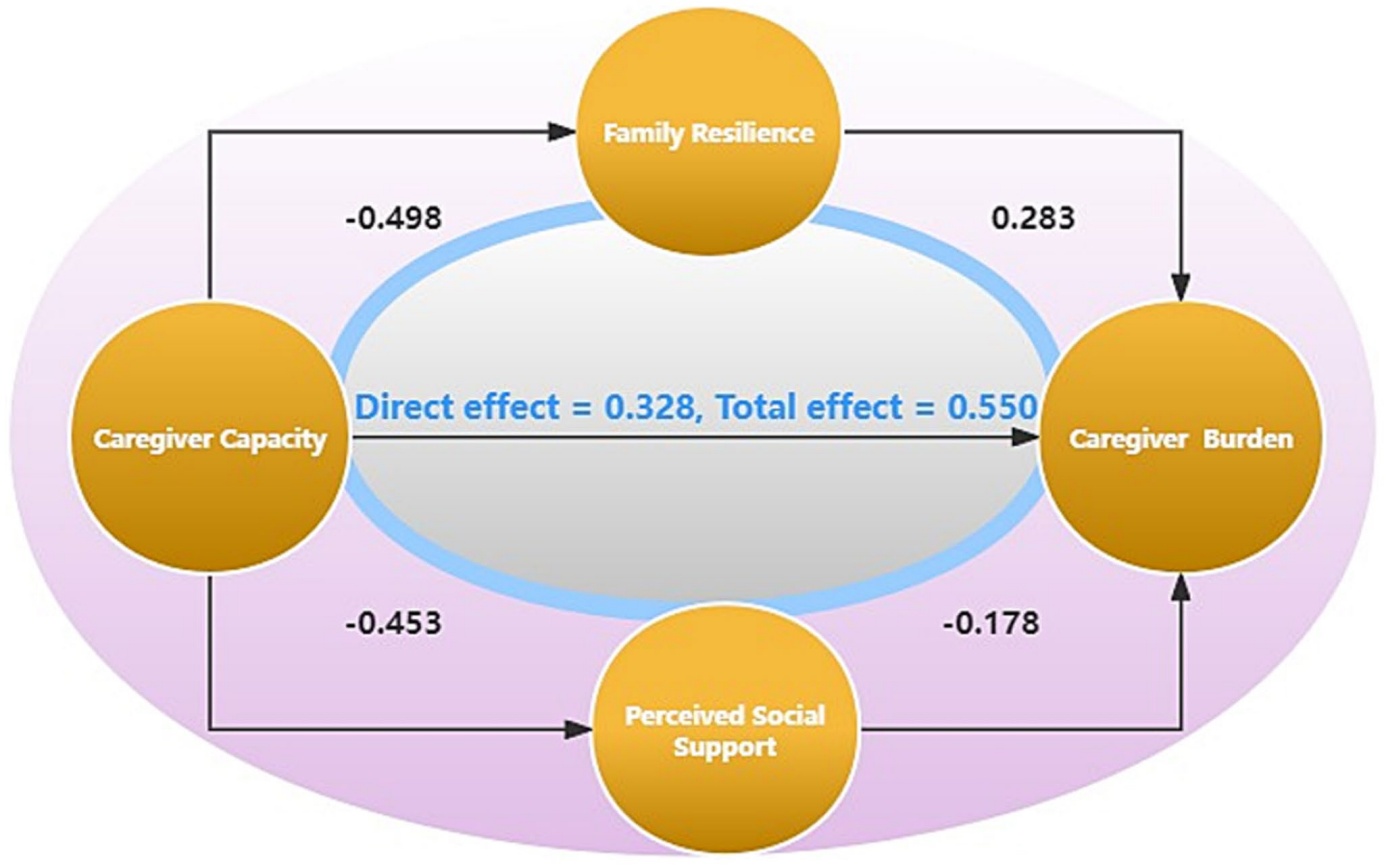
Model of the mediating effect of family resilience\perceived social support on the association between caregiver burden and capacity.

We used Jamovi mediation 2.3.0 to examine the relationships between caregiver burden, family resilience, perceived social support, and caregiver capacity (Table 5; Figure 1). The results indicate that family resilience and perceived social support significantly mediated the association between caregiver capacity and caregiver burden. The indirect effect of caregiver capacity on caregiver burden through family resilience was significant (*β* = 0.141, 95% C.I: 0.091 to 0.176). Similarly, the indirect effect of caregiver capacity on caregiver burden through perceived social support was also significant (*β* = 0.080, 95% C.I: 0.041 to 0.110).

**Table 5:** The mediating effect of family resilience on the association between caregiver capacity and caregiver burden.

				95% C.I. (a)				
Type	Effect	Estimate	SE	Lower	Upper	*β*	*z*	*p*
Indirect	Caregiver burden ⇒ Family resilience ⇒Caregiver capacity	0.13337	0.0217	0.09089	0.17586	0.14117	6.1533	< 0.001
	Caregiver burden ⇒ Perceived social support ⇒Caregiver capacity	0.0758	0.0176	0.04123	0.11037	0.08023	4.2975	< 0.001
Component	Caregiver burden ⇒ Family resilience	−0.29722	0.0248	−0.34588	−0.24855	−0.49839	−11.9704	< 0.001
	Family resilience ⇒ Caregiver capacity	−0.44874	0.0626	−0.57135	−0.32614	−0.28325	−7.1736	< 0.001
	Caregiver burden ⇒ Perceived social support	−0.4221	0.0409	−0.50222	−0.34199	−0.45284	−10.3265	< 0.001
	Perceived social support ⇒ Caregiver capacity	−0.17958	0.038	−0.25406	−0.10511	−0.17718	−4.7262	< 0.001
Direct	Caregiver burden ⇒ Caregiver capacity	0.31014	0.039	0.2337	0.38657	0.32826	7.9524	< 0.001
Total	Caregiver burden ⇒ Caregiver capacity	0.519	0.036	0.449	0.589	0.550	14.550	< 0.001

Furthermore, the component effect analysis revealed that family resilience significantly influenced both caregiver capacity (*β* = −0.498, 95% C.I: −0.346 to −0.248) and caregiver burden (*β* = −0.283, 95% C.I: −0.571 to −0.326). Similarly, perceived social support significantly affected caregiver capacity (*β* = −0.452, 95% C.I: −0.502 to −0.342) and caregiver burden (*β* = −0.177, 95% C.I: −0.254 to −0.105).

Additionally, the direct effect analysis revealed a significant positive association between caregiver capacity and caregiver burden (*β* = 0.328, 95% C.I: 0.234 to 0.386).

## Discussion

### Stroke survivors and caregivers demographics characteristics

The study investigated the factors that contribute to caregiver burden in stroke survivor-caregiver dyads, which included 462 stroke survivor-caregiver dyads. Regarding stroke survivors age, the most significant proportion of stroke survivors fell within the 60–80 age range (37.45%), followed by the 40–60 age range (36.15%). This distribution is consistent with the understanding that stroke predominantly affects older adults, with a higher incidence in individuals over the age of 60.

The level of dependency in ADL was assessed among stroke survivors. The majority of stroke survivors were either partially dependent (36.15%) or moderately dependent (24.24%) on others for ADL. These findings underscore the caregiving responsibilities assumed by the caregivers.

In terms of caregivers, the majority were female (55.41%), which is consistent with the general trend of women assuming the role of primary caregivers in many cultures (Fauziah et al., 2022). Additionally, almost half of the caregivers were children of stroke survivors (49.35%), highlighting the significance of familial relationships in providing care for stroke survivors (Charenkova, 2023).

### Sociodemographic factors influencing caregiver burden

The results of this study suggest that several sociodemographic factors are associated with caregiver burden in stroke survivors. The level of stroke survivors’ dependence on ADL was found to impact caregiver burden significantly. Caregivers of stroke survivors who were partially, moderately, or severely dependent on ADL experienced higher levels of burden compared to caregivers of independent stroke survivors (Ellepola et al., 2022). This finding highlights the increased care demands and challenges faced by caregivers when providing care to stroke survivors with more significant functional limitations.

Additionally, caregivers with a higher education level, such as a bachelor’s degree or higher, reported experiencing higher burdens. This could be due to higher expectations for providing high-quality care. Caregiver health status was found to significantly influence burden, with caregivers in fair or poor health experiencing higher levels of burden. This finding may indicate that caregivers in better health may have greater resilience and coping abilities to manage the demands of caregiving. In this study, we found that caregivers providing more than 8 h of care per day reported lower levels of burden compared to those in the 0–4 h group. This may be due to caregivers with longer hours developing greater adaptation to their roles over time (Vitaliano et al., 2003). However, the impact of caregiver education, health status, and average daily care time needs further research.

### The mediated role of family resilience

Investigations indicate family resilience plays a mediating role between caregiver competence and caregiver burden. The family resilience framework proposed by Walsh emphasizes the significance of adaptive processes and positive family dynamics in overcoming adversity (Walsh, 2003). It indicates that when caregivers possess strong family resilience, they are better equipped to cope with caregiving stress and alleviate their caregiving burden, which aligns with previous studies (Li et al., 2018; Ye et al., 2023). Family resilience promotes positive interactions, improves communication, and enhances problem-solving skills, thereby improving the overall caregiving experience and family well-being (Popescu et al., 2022; Rosenberg et al., 2019). Caregivers with effective communication skills and family adaptability resources are better able to manage sources of caregiving stress (Metze et al., 2015). Our research demonstrates that interventions aimed at enhancing family resilience may serve as an effective way to alleviate caregiver burden and improve their capabilities.

### The mediated role of perceived social support

The results revealed that perceived social support significantly mediated, partially explaining the relationship between caregiver capacity and caregiver burden. The negative correlation between caregiver burden and perceived social support aligns with previous research (Nemcikova et al., 2023; Ong et al., 2018). Caregiving can be an isolating and demanding role, and the availability of social support is essential in buffering the negative impact of burden on caregiver outcomes (Muñoz-Bermejo et al., 2020). When caregivers feel supported by their social networks—whether through family, friends, or community resources—they are more likely to develop resilience against the challenges of caregiving.

Moreover, enhancing perceived social support through interventions—such as support groups or community resources—can significantly improve caregivers’ overall quality of life (Balvert et al., 2022; González-Fraile et al., 2021). By addressing the emotional and practical needs of caregivers, these interventions can reduce their burden and elevate their caregiving abilities, leading to better outcomes for both caregivers and those they care for (Shokrgozar et al., 2022). We recognize and strengthening the role of perceived social support is vital in developing strategies to support caregivers effectively.

### Dual mediation: enhancing caregiver capacity and reducing family burden in Chinese context

The research findings suggest a double mediation effect in the relationship between caregiver capacity and caregiver burden in Chinese families. This means that family resilience and perceived social support play significant roles in mediating this relationship. These results have important implications for developing interventions and support systems targeting individual and family-level resources to enhance caregiver capacity and reduce burden.

In Chinese culture, Confucianism’s influence emphasizes filial piety and respect for elders, where family caregiving is prevalent. The emphasis on family obligations and respecting elders motivates Chinese caregivers to provide the best care possible for their aging parents or relatives (Qiu et al., 2018). The government in China has also promoted the use of internet hospitals, combining online consultations with the home care model to facilitate caregiving. By targeting family resilience and social support, interventions can help caregivers build coping strategies, enhance their well-being, and improve their caregiving capacity.

### Limitations

We acknowledge several limitations in our study. First, the sample was drawn from a single public hospital in Ningbo, China, which may restrict the generalizability of our findings to other regions and healthcare settings. Second, we did not collect data on stroke severity, which hinders our understanding of the population and its relevance to other stroke services. Additionally, the reliance on self-reported measures can introduce response bias and social desirability effects, potentially compromising data validity. We also excluded participants with a history of depression or those using antidepressant medications, which may influence the results. Assessments conducted by a nurse while the stroke survivor was still hospitalized may not capture the full range of challenges caregivers face once they return home. The Barthel ADL Index may not fully reflect the patient’s actual functional status, particularly in cases of apraxia or cognitive impairments.

Moreover, although our findings support the proposed mediation model, this study did not explore other potential mediating and moderating variables. Furthermore, the relationship between caregiver burden and capacity is likely bidirectional, underscoring the need for future research to consider additional influencing factors and the dynamic interplay between caregiver burden and capacity.

## Conclusion

In conclusion, this study sheds light on the dual mediation effect in the relationship between caregiver capacity and caregiver burden in Chinese families. The findings highlight the importance of considering family resilience and perceived social support as significant factors in mitigating caregiver burden and enhancing caregiver capacity. By acknowledging and addressing the dual mediation effect, policymakers, healthcare professionals, and support organizations can work together to create comprehensive and culturally sensitive strategies that alleviate caregiver burden and enhance caregiver capacity in Chinese families. Ultimately, this will improve caregivers’ well-being and care outcomes for stroke survivors in China.

## Data Availability

The raw data supporting the conclusions of this article will be made available by the authors upon reasonable request.
